# Falsely diagnosed thyrotoxicosis caused by anti-streptavidin antibodies and pre-wash procedures

**DOI:** 10.1186/s13044-021-00108-y

**Published:** 2021-07-10

**Authors:** Takuya Ishikawa, Hiroyuki Sakai, Tokutaro Itaya, Suwanai Hirotsugu, Jumpei Shikuma, Takashi Miwa, Ryo Suzuki, Masato Odawara

**Affiliations:** Department of Diabetes, Metabolism and Endocrinology, Tokyo Medical University, 6-7-1 Nishishinjuku Shinjuku-ku, Tokyo, 160-0023 Japan

**Keywords:** Immunoassay interference, Thyroid function tests, Anti-streptavidin antibody, Pre-wash

## Abstract

**Background:**

Anti-streptavidin antibodies are causal determinants of analytical interference during Thyroid function tests, and numerous reports have detailed such interference, with anti-streptavidin antibodies attracting attention.

**Case presentation:**

We conducted a straightforward investigation of interference due to anti-streptavidin antibodies, with a case of a 60-year-old Japanese man who consulted our department for inconsistencies between his clinical course and Thyroid function tests. Experiments were conducted using Cobas8000 e602, which employs assay procedures with pre-wash to evaluate FT4 and FT3 levels.

**Conclusions:**

To our knowledge, this is the first published report to clearly investigate such interferences using a combination of polyethylene glycol precipitation, heterophilic blocking tube precipitation, streptavidin-coated magnetic particle precipitation, and different instruments with or without pre-wash. Clinicians should consider that interferences caused by anti-streptavidin antibodies could lead to a misdiagnosis of thyrotoxicosis. Moreover, discussions between laboratory specialists, clinicians, and manufacturers are required to identify interferences and avoid unnecessary examinations and inappropriate treatment.

## Background

Typical thyroid function tests (TFTs) usually evaluate thyrotropin (TSH), free thyroxine (FT4), and free triiodothyronine (FT3) levels [1]. Anti-streptavidin antibodies are causal determinants of analytical interference during TFTs [2, 3], and numerous reports have detailed such interference, with anti-streptavidin antibodies attracting attention [1, 4–7]. We conducted a straightforward investigation of interference due to anti-streptavidin antibodies during TFTs. Experiments were conducted using Cobas8000 e602, which employs assay procedures with pre-wash to evaluate FT4 and FT3 levels. All the measurements for FT4 were examined using FT4 II reagent while measurements of FT3 were examined with FT3 III reagent.

## Case presentation

We report a case of a 60-year-old Japanese man who consulted our department for inconsistencies between his clinical course and TFTs. He had undergone right subtotal thyroidectomy for papillary thyroid carcinoma when he was 57 years old. After surgery, levothyroxine replacement at 100 μg/day was used to control his thyroid hormone levels. For over 2 years, TFTs revealed normal TSH, FT4, and FT3 levels. However, 28 months after thyroidectomy, abrupt changes in TSH (0.05 μIU/mL), FT4 (3.24 ng/dL), and FT3 (7.00 pg/mL) were observed (each reference range is shown in Tables 1 and 2). Anti-TSH receptor antibodies (TRAb) were slightly positive (2.1 IU/L; reference range: < 2.0 IU/L). Subsequently, the surgeon established a diagnosis of thyrotoxicosis and discontinued levothyroxine. Since the TFT results did not improve, the surgeon started potassium iodide treatment. Nevertheless, the TFTs still revealed a pattern of syndrome of inappropriate secretion of TSH (SITSH) (Table 1, before polyethylene glycol (PEG) precipitation (Tx)), which prompted consultation with our department. Upon consultation, the patient presented with no typical symptoms of thyrotoxicosis. He did not take any biotin related supplments. Therefore, considering the possibility of a false SITSH, PEG Tx using the same sera was performed.

**Table 1:** Detection and characterization of interference using polyethylene glycol, heterophilic blocking tube, and streptavidin-coated magnetic particle SA-MP treatment

	TSH, μIU/mL (RR: 0.50–5.00)	FT4, ng/dL (RR: 0.90–1.70)	FT3, pg/mL (RR: 2.3–4.0)
Patient			
Before PEG Tx	4.67	2.66	5.88
After PEG Tx (actual measured value)	4.77	0.87	2.11
After PEG Tx (calculated value)	9.54	**–**	**–**
	TSH	FT4	FT3
Recovery (%)	204 (RR: 30–80)	33 (RR: 60–90)	36 (RR: 60–90)
	TSH, μIU/mL (RR: 0.50–5.00)	FT4, ng/dL (RR: 0.90–1.70)	FT3, pg/mL (RR: 2.3–4.0)
Patient			
Before HBT Tx or SA-MP Tx	4.36	2.15	4.27
After HBT Tx	6.49	1.68	3.86
After SA-MP Tx	9.81	1.04	2.7
Control			
Before HBT Tx and SA-MP Tx	3.65	1.63	3.62
After HBT Tx	3.69	1.64	3.64
After SA-MP Tx	3.64	1.66	3.61

PEG Tx resulted in a high TSH level but low FT4 and FT3 recovery. HBT Tx or SA-MP Tx resulted in a higher TSH but lower FT4 and FT3 values. However, HBT Tx or SA-MP Tx showed almost the same results in the control sample

**Table 2:** Thyroid function test parameters as measured by different immunoassays (Elecsys and Architect)

	Elecsys		Architect
	Cobas8000 e602 (with pre-wash)	Cobas e411(without pre-wash)	
Patient			
TSH, μIU/mL	4.36 (RR: 0.50–5.00)	4.77 (RR: 0.50–5.00)	8.57 (RR: 0.35–4.94)
FT4, ng/dL	2.15 (RR: 0.90–1.70)	1.31 (RR: 0.90–1.70)	0.86 (RR: 0.70–1.48)
FT3, pg/mL	4.27 (RR: 2.3–4.0)	3.66 (RR: 2.3–4.0)	2.44 (RR: 1.71–3.71)

Cobas8000 e602, which is performed with pre-washing, resulted in almost the same level of TSH and with higher FT4 and FT3 values compared to Cobas e411, which is not performed with pre-wash. Architect provided the highest TSH level and lowest FT4 and FT3 values among the three instruments

PEG Tx was performed following previously described procedures [8, 9]. PEG Tx promoted high levels of TSH but low FT3 and FT4 recovery. Subsequently, Heterophilic Blocking Tube (HBT) (Scantibodies Laboratory, Santee, CA, USA) Tx was performed to remove heterophilic antibodies. HBT Tx results thereafter showed that the patient’s sample recorded higher levels of TSH and lower FT3 and FT4 recovery compared to those of the control samples. Furthermore, two additional assays were subsequently performed: (1) the Elecsys assay using Cobas e411, which employs the same method as Cobas8000 e602 except for the pre-wash procedure, and (2) The two-step method, Architect assay, which utilizes a completely different method than the one-step Elecsys assay.

Evaluating the thyroid function using the Architect method resulted in a higher TSH level and lower FT4 and FT3 values compared with the Elecsys method. Since Cobas e411 is not performed with a pre-wash, Cobas e411 was used to compare the possible effects of pre-wash in the thyroid function evaluation. As such, both FT4 and FT3 values were lower with Cobas e411 than with Cobas8000 e602. Nonetheless, the TSH values measured using Elecsys, which does not include pre-wash, produced similar results (shown in Table 2). We further examined whether anti-streptavidin antibodies interfere with TFTs by adding streptavidin-coated magnetic particles (SA-MP) (shown in Table 1). The experiment showed higher TSH and lower FT3 and FT4 recovery in the patient’s sample. We consequently concluded that interference was caused by the streptavidin antibodies.

One year after consultation, false values of the TFTs were gradually improving, so the patient resumed the original with levothyroxine treatment at 100 μg/day. (TSH: 1.60μIU/mL, FT3: 3.05 pg/mL, FT4: 1.39 ng/dL).

## Discussion and conclusions

Of note, Lam et al. have identified two cases of IgM anti-streptavidin antibodies causing analytic interference during TFT [4], and Verougstraete et al. have implicated IgM (rather than IgG) in such interference [10]. The relatively brief period (~ 1 year) during which our patient was plagued by false values is further evidence that anti-streptavidin antibodies are likely IgM type. Indeed, several case reports have indicated falsely increased and decreased values for TFTs due to anti-streptavidin antibodies. However, to our knowledge, this is the first published report to clearly investigate such interferences using a combination of PEG Tx, HBT Tx, SA-MP Tx, and different instruments with or without pre-wash. Our experiment revealed that pre-wash probably increased measurement error during TFTs. Given that Cobas8000 e602 performs B/F separation by pre-wash before B/F separation in the measuring cell, non-specific reactions due to anti-streptavidin antibodies increased more with Cobas8000 e602 than with Cobas e411. Altogether, the anti-streptavidin antibodies in the patient’s serum interacted with streptavidin in the assay reagents, thus leading to a reduced luminescence signal and falsely increased FT4 and FT3 values with a competitive immunoassay. Additionally, more attention should be placed on the finding that TRAb values returned to the reference value within 1 year (1.0 IU/L). The Elecsys TRAb competitive assay similarly tends to produce falsely elevated values in the presence of anti-streptavidin antibodies. We are unable to offer corroboration, because TRAb was not investigated in tandem with FT4 and FT3 on this occasion. Still, cases of thyrotoxicosis ascribed to anti-streptavidin antibodies have been linked to falsely high values of TRAb in past reports [1.4.7]. The clinical course of such current patient might be misdiagnosed of thyrotoxicosis due to Graves’ disease, and the patient might have seemed to be cured within 1 year through unnecessary antithyroid drugs unless we had suspected interference due to anti-streptavidin antibodies. Studies have shown that interference due to anti-streptavidin antibodies is not rare [10]. Clinicians should consider that interferences caused by IgM anti-streptavidin antibodies could lead to a misdiagnosis of thyrotoxicosis. With such uncertain cases, we recommend that PEG Tx be performed to determine the potential for interference. Recently, the Elecsys kit improved its reagent for FT4, which reduces interference by anti-streptavidin antibodies. However, reagents for TSH and FT3 have not been improved yet. Therefore, discussions between laboratory specialists, clinicians, and manufacturers are required to identify interferences and avoid unnecessary examinations and inappropriate treatment. In conclusion, through the combination of PEG Tx, HBT Tx, SA-MP Tx, and other measurement methods, we showed that anti-streptavidin antibodies caused interference during TFTs.

## Data Availability

The datasets used and/or analyzed during the present study are available from the corresponding author upon reasonable request.

## Abbreviations

TFTs: Typical thyroid function tests
TSH: Thyrotropin
FT4: Free thyroxine
FT3: Free triiodothyronine
TRAb: Anti-TSH receptor antibodies
SITSH: Syndrome of inappropriate secretion of TSH
PEG: Polyethylene glycol
Tx: Precipitation
HBT: Heterophilic Blocking Tube
RR: Reference range
SA-MP: Streptavidin-coated magnetic particles
